# History of Anesthesia and Pain in Old Iranian Texts

**DOI:** 10.5812/aapm.15363

**Published:** 2014-06-23

**Authors:** Ali Dabbagh, Samira Rajaei, Samad E J Golzari

**Affiliations:** 1 Anesthesiology Research Center, Shahid Beheshti University of Medical Sciences, Tehran, Iran; 2 Department of Immunology, School of Medicine, Tehran University of Medical Sciences, Tehran, Iran; 3 Department of Anesthesiology, Tabriz University of Medical Sciences, Tabriz, Iran

**Keywords:** History, Anesthesia, Pain

There are many references in ancient Iranian literature on anesthesia, analgesia and pain. Various cultural changes have occurred due to political, cultural and civil factors, one key example being the bilateral interactions between Iranian culture and other cultures with regards to many issues, involving medicine and medical sciences, a process that has been part of the dialogue between civilizations. Some aspects of ancient Iranian culture have been cited in Iranian literature, often involving Iranian physicians. Iranian scientists of earlier times possessed a great wealth of knowledge in the fields of medicine, philosophy, literature, astrology, etc. and they recorded their experiences for posterity. Ancient Iranian culture cites the writings of many famous and great scientists and poets. Two main cultural eras are defined in Iranian culture: pre-Islamic and Islamic. In this manuscript, the recorded histories of anesthesia, analgesia and pain are followed according to this historical classification. Throughout the manuscript pain is frequently mentioned, because pain is one of the key issues often mentioned in Iranian literature, especially by the romantic poets, mainly as a sign of ‘love’ from the ‘lover’ expressed towards the ‘beloved one’. Lover in Farsi is A'ashegh and the beloved one is Ma'shough; two terms that will be mentioned throughout this manuscript.

## 1. Anesthesia, Analgesia and Pain in The Pre-Islamic Era of Iranian Culture

The text of Shahnameh appears to be a proof of the fact that general anesthesia had at least been described in ancient Farsi texts and possibly, that in ancient Iran general anesthesia was used in surgical procedures for the first time. This is described below. In 550-330 BC, under Cyrus and Darius, two kings of the Achaemenid Empire, the Iranian Empire eventually became the largest and most powerful empire in human history to that point in time (1-3). This empire stretched from the Indus and Oxus rivers in the east, to the Mediterranean sea in the west, it also extended through Anatolia (part of modern-day Turkey) to modern-day Egypt. The most important interactions of Iranian culture in the pre-Islamic era were with the ancient Greek empire, since they were the two main competing cultures and empires of the time. On occasion, these interactions even took the form of bloody wars. The main perspective of Iranian culture was concerning the key humanistic approaches, influencing all community life aspects. This is presented as the first human rights declaration on the Cyrus Cylinder, now preserved in the British Museum of London. It contains ‘references to justice and peaceful rules’ from around 550 BC. Therefore, Cyrus Cylinder is believed to be ‘an early charter of human rights’ (4). Large areas of Iranian science (including medical texts) came under the aegis of the ancient Greek Empire, following the Persepolis invasion by Alexander (1-4) in 334 BC, which led to the end of the Achaemenid Empire. Ancient Greek culture had made an important contribution to anesthesia and related issues. It has been documented that in ancient times in the pre-Islamic era, Indian and Persian surgeons were very much respected and had operating skills regarding plastic nasal surgery and cataract couching (1-3). The anesthetic techniques used in this era are mentioned in texts written around 1000 years later, mainly by Ferdowsi; a combination of cannabis and camphor used at the birth of Rostam (which was prepared as a special wine, given to Roudabeh, the birth giving mother, by a Zoroastrian), also recorded as the first citation of an alternative method of childbirth, called Rostam-zad, described below. Although Shahnameh is a narrative text, according to Ferdowsi its stories are based on the actual lives of the kings. However, this section of Shâhnameh appears to be a proof of the idea that general anesthesia had at least been described in ancient Farsi texts. This issue is described further in the following pages, under Ferdowsi in the Islamic era (4). Medicine has a very long and interesting history in ancient Iran. Some ancient academic centers like Jundi Shapur still exist in Iran (established in the third century AD). These universities provided a creative environment for scientific research and cooperation between scientists from different cultures, extending throughout the Islamic era. A key point is that ‘the credit for the whole hospital system’ is principally due to the ancient Iran (1-4). Shahr-e Sukhte (Burnt City), known as the most important city of prehistoric Iran, dating from the third millennium BC, is located in the southeast of the country. Archaeologists there have found a skull that anthropologists believe to be ‘the first evidence’ of brain surgeryperformance, a procedure impossible to carry out without anesthesia, from the prehistoric Iran (1-3); the skull of a 13year-old girl, diagnosed posthumously with chronic hydrocephalus (1).

## 2. Anesthesia, Analgesia and Pain in the Islamic Era of Iranian Culture

The Islamic era is one with expanded cross-cultural interactions. Islam was introduced to Iran by Arab people around 14 centuries ago. During the spread of Islam from the Arabian Peninsula throughout the other countries, like Egypt and Iran, Islamic culture encountered ‘long established civilizations and centers of learning’. Arabian scientists translated scientific texts from the original languages, into Arabic (5). After acceptance of Islam, Iranians experienced many changes in the fields of language, culture and science. Many scientists flourished, following the teachings of Islam that encouraged science, serving others and saving lives, as valued criteria (6). ‘Whoever resuscitates a human life (his work) is exactly the same as he has resuscitated the whole mankind’ is an instance from what the Quran says, regarding the mentioned subjects. (Chapter 5, Verse 32). This citation is clear about the invaluable role of resuscitation.There is one very important point to be mentioned here, before citing the key scientists and poets: the role of the geographical map in relation to ancient Iran. As we know, the current national and political geography of the countries, recognized by the nations and their governments, differs from that in existence many centuries before. The ancient Iranian empire borders have been considerably extended, both eastwards and westwards, from the time of the Achaemenid Empire and over many centuries throughout the Islamic era (7). The Persian Empire was founded around 550 BC by Cyrus II and extended by Darius I, therefore, while the scientists and poets from ancient times may be described as Iranian, the cities they inhabited may not be within the modern-day borders of Iran. This is also the case with ancient Greek and Roman cities. A number of Mediterranean cities in various countries, according to modern geographical and political maps, were previously under Greek jurisdiction. However, for the purposes of this manuscript we will not discriminate on such grounds. Fortunately, a number of other nations and countries developed from the divided Persian territory and share the common bond of an association with the cultural and scientific heritage of the ancient Iran (8). The development of teaching hospitals, modeled with patterns similar to modern medical schools and research activities in the field of biomedicine, first originated from the cooperation of scientists from different cultures at Jundi Shapur University Hospital, located in the modern-day Iranian region of Khuzestan, during the Sasanian Empire, in pre-Islamic Iran, eventually led to further progress during the golden age of Islam, creating the foundations for modern academic medicine. According to the documents available from that period, it is possible that certain physicians in the Islamic era were able to perform some of the steps of inhalational anesthesia, much of this was performed at Jundi Shapur University Hospital, which had the necessary facilities (1, 7, 8). In this part of the manuscript, texts related to anesthesia or pain, as recorded by some of the most famous Iranian poets and scientists are briefly presented. Ferdowsi, Avicenna, Rhazes, Jorjani, Hafez, Molavi, Sa'adi and Khayyam may be regarded as being the cornerstones of the Iranian culture, during the Islamic era.

## 3. Ferdowsi

Ferdowsi is one of the greatest Iranian poets, he wrote his masterpiece Shâhnameh (The History of the Kings) around 1000 AD. In this book, he talks about Iran’s past both mythically and historically, before and until the 7th century, the period when Islam was introduced to Iran. In the stories of Shahnameh the use of medicine, including that of the poppy (used orally for pain relief and creating a sense of well-being), has been described. One of the heroes of Shahnamehis Rostam, who was the son of Zaal. Nariman, the grandfather of Zaal, was a special guardian of the country and an ancient Iranian hero: in Shahnameh the country is named ‘Iran-zamin’ [the land of Iran) (1, 4). Zaal’s wife had a very difficult childbirth with Rostam, following which Zaal burned one of the three remaining feathers of Simorgh (the mythical bird in Shâhnameh, related to the Greek Sphinx), which then led to the appearance of Simorgh, who created a novel method of delivering a baby via the abdomen, which many decades later became known as a caesarean section (‘Rostam-zad’ in Shahnameh). Zaal used a combination of cannabis and camphor to ameliorate the surgical pain (4). In another section of Shâhnameh, the adult Rostam becomes the chief guardian of the country. In one of the greatest and best-known passages in Shâhnameh, Rostam quarrels with his son, Sohrab, failing to recognize each other after long living apart. Following this lengthy quarrel, Rostam eventually kills Sohrab, but on realizing that his adversary is in fact his son, tries to treat and save him. He asks the king to send a pain relief and a life-saving antidote (‘Noosh-Daru’), but the medications arrive too late to save Sohrab and he dies. Rostam, although grief-stricken, saves his country (4). In ShahnamehFerdowsi tells us more about real somatic pain and its treatments, rather than the pain love (4).

## 4. Avicenna (Aka Pouresina or IbnSina)

Avicenna, who lived in the 10th and 11th centuries, is well known as the father of modern medicine. He was not only a physician, also an Islamic scholar, psychologist and theologian, an astronomer, a chemist, a geologist, a physicist, a teacher, a paleontologist, a logician, a mathematician and a poet. Avicenna is known to be the most influential and famous scientist from the golden age of the Islamic era. Avicenna’s tomb is located in Hamadan, a city in northwestern Iran. Many citations state that both Avicenna and Rhazes were Iranians (9-14). A number of Western researchers also believe that Avicenna was born in Persia and that his most famous text, translated as Canons of Medicine, was the main medical textbook in Persia and Europe for over 500 years (15-18). Avicenna adopted the theory of epidemics from the Greeks. The Canon of Medicine was the first text to describe experimental medicine, including evidence-based medicine, randomized controlled trials and related issues. In addition, he established seven basic rules for testing the effectiveness of new drugs and medications, leading to a modern basis for clinical pharmacology and medical trials (19-21). The scientific citations attributed to Avicenna include the following techniques: tracheotomy, pharyngeal intubation and a method for clearing the secretions of the upper airway in stridor and respiratory distress. He also discovered and described a number of plants having pharmacological effects, like opium; and introduced a number of methods for anesthesia induction and surgical analgesia implementation (22, 23). His recommended analgesic techniques included drug-impregnated sponges and compresses, ointments, oils, aroma therapeutic salts and drinks, smoke, pills and many more. These methods of analgesia demonstrate not only the depth of knowledge of Avicenna, but also his ability to put that knowledge into practice. Analgesia-related references to Avicenna are numerous. He wrote of three pain-alleviating groups of drugs:

1. those that counteract and nullify pain, like fennel or linseed, used directly as a poultice on the painful region;
2. those that induce sleep and decrease activity levels, like inebriants, milk and sweet water; and
3. those that decrease the perception of pain, like narcotics and somniferous agents (24).

Avicenna also described a method for rendering a person unconscious and hence oblivious to pain, using opium, henbane and mandrake. Avicenna listed 15 different types of pain: boring, compressing, corrosive, dull, fatigue, heavy, incisive, irritant, itching, pricking, relaxing, stabbing, tearing, tension and throbbing (9, 10).

## 5. Rhazes

Rhazes, who lived in the 9th and 10th centuries, was a Persian physician, chemist and scholar and also a great medical teacher. He was born and died in Rey, near Tehran, the modern-day capital of Iran (11, 12). Some believe he was possibly ‘the greatest clinician of all time’ (21-22). Many ‘firsts’ in chemistry, medicine and medical research are considered to be among his discoveries or are attributed to his writings, including smallpox, measles, alcohol and many discoveries in the field of chemistry (11, 12). His writings are recorded in over 200 books and articles. He is considered the first physician to have used opium for anesthesia and was also a great neurosurgeon, having described his methods for the diagnosis and treatment of hydrocephalus (13). He was not only the first to discover alcohol, he also used it for the first time in the field of medicine. His writings were frequently cited later in European texts (5, 11-14, 20-22, 24,25).

## 6. Jorjani

Esmail Jorjani was a Persian physician and anatomist who lived in the 11th and 12th centuries. He wrote a comprehensive textbook of medicine, Zakhire-ye Kharazmshahi (The Treasure of King Kharazm), considered being the oldest Persian medical textbook (11). Among the many topics in this book, one might mention his comprehensive description of the cranial nerves, in which he gave a comprehensive explanation of trigeminal neuralgia, hemifacial spasm and Bell's palsy (5, 12, 13). Some believe he was the first to recognize ‘artery-nerve conflict’, as one of the causal factors of trigeminal neuralgia, documented in Zakhire-ye Kharazmshahi. Additionally, some believe that this theory was later adopted by Dandy and Jannetta, as a part of their theory for the modern surgical approach used in trigeminal neuralgia. Among the other interesting topics covered by Jorjani in this book, regarding pain, are his writings on obstetrical pain and the complications of childbirth (5, 11-14,20-22, 24,25).

## 7. Molavi

Molavi (Jalal-ad-Din Mohammad Balkhi, aka Mowlana or Rumi) was a Persian Muslim poet and an Islamic theologian, living in the 13th century. The location of his birth and the native language used throughout his various texts, indicate that he was of Persian heritage. He was born in either Wakhsh or Balkh (then both located in Khorasan, a province of Iran, but now in modern-day Tajikestan and Afghanistan, respectively). He was buried at Konya (in modern-day Turkey). The majority of his books and nearly all of his poems have been published in modern Farsi. He is cited many times with regard to pain and its alleviation through the use of opium and wine, especially in his poems on Sufism. His writings are too numerous, extensive and broad-ranging to be summarized in this article (14).

## 8. Sadi

The following well-known verse written by the poet Sa'adi can be found as a general humane concept among all the human being: The sons of Adam are organs of the same body; for they are created from the same essence. Should an organ be afflicted by pain of the era, the other organs would not be calm and silent if you disregard other people's sorrowfulness you are not eligible to be called a human being. Sa'adi was a very famous Iranian poet who lived in the 12th and 13th centuries in Shiraz, one of the main cities of ancient and modern Iran. In Iranian culture and literature, the writings of Sa’adi are among the best known. Much of his writing was in the form of poetry or of prose, containing poetry. His fictional work embraced the topics of pain, anesthesia and medical ethics (25).

## 9. Hafez

Hafez, one of the greatest Farsi-speaking poets, lived in Shiraz in the 14th century. In his romantic poems, he frequently documented the pain which the lover (A'ashegh) experiences on account of the distance, separating him from his beloved (Ma'shough). Since the lover wishes and hopes to be reunited with his beloved, he believes that his suffering (pain) is not intolerable and does not wish it to be treated, in order that she will never be out of his thoughts. Hafez wrote:

It is better for me to have my pain hidden from interfering physicians,would it be that I get treated by the hidden treasures (of God). He has many other poems regarding pain and anesthesia (5).

## 10. Khayyam

Khayyam was a poet, mathematician, philosopher, musician, astrologist and Islamic scholar, living in the 11th and 12th centuries. He wrote a number of scientific and literary texts on many subjects, including mathematics, algebra and calculations. He also prepared a revised version of a calendar, still very accurate today. His poems are in the form of quatrains (originally called Rubaeiyat), in which he frequently makes reference to pain and its alleviation by opium and wine. One of the best known translations of his book of poems into English was done byEdward Fitzgerald, in the 19th century (26).

## 11. Others

There many other remarkable texts by ancient Persian physicians and writers, citing the Iranian physicians quotations, among which are so many poets, making it hard to mention all, individually. They have written in Farsi on anesthesia, pain and analgesia, in the ancient Persian texts, under the umbrella of Islamic medicine, Islamic Science and Islamic knowledge (5, 20-22,24).
